# Stem Cell Therapy for Stress Urinary Incontinence: Why Has Clinical Success Remained Limited?

**DOI:** 10.1007/s00192-026-06830-1

**Published:** 2026-08-22

**Authors:** Rodrigo A. Castro

**Affiliations:** Department of Urogynecology and Vaginal Surgery, Federal University of São Paulo (UNIFESP/EPM), São Paulo, Brazil

**Keywords:** Intrinsic sphincter deficiency, Mesenchymal stromal cells, Midurethral sling, Regenerative medicine, Stem cell therapy, Stress urinary incontinence

## Abstract

**Introduction and Hypothesis:**

Stem cell therapy for stress urinary incontinence (SUI) has been pursued for more than a decade, supported by a compelling regenerative rationale and reassuring early-phase safety data. Yet clinical efficacy has remained modest, complete continence uncommon, and durability uncertain.

**Method:**

In this Clinical Opinion, we argue that translation has stalled for reasons that are both biological and conceptual: heterogeneous cell products and manufacturing variability undermine reproducibility; endpoints and follow-up are insufficiently harmonized to benchmark against surgical standards; chronically fibrotic periurethral tissue may be biologically quiescent and poorly permissive for regenerative activation; and, fundamentally, continence is a biomechanical phenomenon that cell injections alone cannot replicate.

**Results:**

We propose that future progress will depend on validated potency assays, mechanism-driven trial design, microenvironment priming strategies, and integration of regenerative biology with biomechanical reinforcement (e.g., scaffolds or biologically enhanced support constructs).

**Conclusion:**

Until such integration occurs, stem cell therapy for SUI should remain investigational.

## Introduction: Why Regeneration Still Attracts Clinicians

Stress urinary incontinence (SUI) is defined as involuntary leakage on effort or physical exertion, or on sneezing or coughing [1]. It remains one of the most prevalent pelvic floor disorders worldwide and a major contributor to impaired quality of life, social restriction, reduced physical activity, and health care utilization [2]. The pathophysiology is multifactorial: urethral hypermobility and intrinsic sphincter deficiency (ISD) often coexist, compounded by childbirth-related neuromuscular injury, age-associated changes in urethral vascularity and connective tissue, and progressive extracellular matrix remodeling.

Contemporary management is well established. Pelvic floor muscle training is the first-line intervention, whereas midurethral sling surgery provides durable and reproducible outcomes with strong evidence from systematic review and long-term follow-up literature [3, 4]. Nonetheless, surgery is not universally acceptable. Concerns about complications, mesh controversies, recurrence, and patient preference for less invasive approaches continue to drive interest in alternative strategies. Regenerative medicine—particularly stem cell–based therapy—has emerged as an intuitively appealing concept: restore the damaged continence mechanism rather than compensate for it mechanically.

Yet, despite more than a decade of clinical experimentation, stem cell therapy has not become practice changing. Early trials have repeatedly demonstrated feasibility and reassuring short-term safety [5–7]. Improvements in pad test weight, cough stress testing, and symptom scores have been reported in subsets of patients, suggesting biological activity. However, effect sizes are generally modest, complete continence is uncommon, and durability beyond short- to mid-term follow-up remains uncertain [6, 8, 9]. In contrast to established surgical standards, outcomes have not achieved consistent reproducibility or magnitude of benefit. This persistent gap between promise and performance warrants a careful, mechanism-focused appraisal.

## Mechanistic Biology: From Replacement to Context-Dependent Signaling

Early regenerative models implicitly adopted a “replacement” paradigm: injected myogenic progenitors would engraft into the urethral sphincter and regenerate striated muscle, restoring contractile strength and closure pressure. The earliest human studies largely used autologous muscle-derived cell preparations and were framed around this myogenic rationale [5–7]. However, across regenerative medicine, the mechanistic understanding of mesenchymal stromal cells (MSCs) has evolved substantially. Genetic and senescence are important considerations for clinical-grade MSC manufacturing [10–13]. A growing consensus holds that, for MSC products, durable structural engraftment is limited and the dominant mechanism is paracrine and trophic modulation rather than architectural replacement [14, 15].

Caplan’s proposal to reconsider MSC nomenclature—emphasizing “medicinal signaling” rather than stemness—captured a key translational insight: MSCs behave as environmentally responsive therapeutic platforms whose effects depend on the host microenvironment [11, 14]. The MSC secretome includes cytokines and growth factors (e.g., VEGF, HGF), immunoregulatory mediators (e.g., IL-10), extracellular vesicles and exosomes, and matrix-associated enzymes that can influence angiogenesis, inflammation, apoptosis, oxidative stress, and extracellular matrix (ECM) remodeling [14, 16]. Importantly, MSCs exhibit immunomodulatory plasticity: their functional phenotype can shift depending on microenvironmental cues, a property described as “licensing” or activation-dependent functionality [12].

This context dependence is critical when translating to SUI. MSC activity is strongest in environments characterized by acute injury or active inflammation, where cytokine and chemokine signaling provides the stimuli required for robust trophic cascades. In contrast, periurethral tissues in chronic SUI often reflect long-standing remodeling: fibrosis, altered collagen architecture, reduced vascularity, and neuromuscular attenuation. Such a niche may be biologically quiescent and therefore insufficiently permissive for MSC activation and sustained remodeling [8]. Injection-related microtrauma is unlikely to recreate the complex inflammatory signaling of acute injury models, limiting the magnitude and durability of repair responses.

Additional mechanistic constraints involve cell retention and survival. After periurethral injection, a substantial proportion of delivered cells may be rapidly lost owing to mechanical dispersion, ischemia, apoptosis (including anoikis), and immune clearance depending on product characteristics [10]. Even when early detection of labeled cells is feasible, convincing evidence of durable structural integration in human tissue remains limited. Consequently, the biological effect is increasingly thought to be mediated primarily through paracrine signaling rather than long-term tissue replacement. Clinically, this is consistent with the pattern observed in many trials, where early symptomatic improvement occurs but a durable cure remains uncommon.

An under-discussed but clinically relevant issue is dosing biology. If the dominant mechanism is transient paracrine signaling, then the “dose” is not only the cell number injected but also the duration of secretome exposure and spatial distribution within the urethral complex. A single periurethral injection may provide a short-lived signaling pulse. This raises the possibility that repeated dosing, sustained-release carriers, or vesicle-based products may better match the time course required for meaningful remodeling [9, 11, 14, 17]

Finally, donor biology and cellular senescence are not trivial. Genetic stability and senescence are key considerations for clinical-grade MSC manufacturing [10]. Autologous cells obtained from older patients—who represent a substantial proportion of the SUI population—may exhibit reduced proliferative capacity, altered secretory profiles, and increased senescence markers that can diminish regenerative potential and may even bias signaling toward pro-fibrotic pathways [10, 15]. This “autologous paradox” implies that patients most likely to seek nonsurgical options may also be those least likely to yield potent autologous cell products.

## Clinical Evidence: Feasibility Confirmed, Efficacy Still Limited

Clinical trials in SUI have consistently demonstrated that periurethral injection of autologous muscle-derived cells is feasible and, in the short term, appears safe [5–7]. Dose-ranging work has also suggested biological activity, with some patients experiencing improvements in pad test weight, stress test results, and patient-reported outcomes [6]. Nevertheless, three recurring observations limit clinical enthusiasm: complete continence is uncommon, response heterogeneity is substantial, and durability remains uncertain, largely owing to limited follow-up and variable endpoint definitions [8, 18].

The contrast to midurethral sling surgery is instructive. Sling efficacy is supported by a high-quality evidence base and long-term outcome data [3, 19]. If a regenerative therapy is to compete clinically, it must demonstrate not only safety but also clinically meaningful effect sizes and durability comparable with established standards. To date, stem cell therapy has not reached this bar. This does not negate biological plausibility; rather, it suggests that either the delivered biological effect is insufficient for the mechanical demands of continence or the therapeutic implementation (product definition, delivery, patient selection, outcome measurement) remains suboptimal.

A practical challenge is that “stem cell therapy” is often discussed as a unitary intervention, whereas the clinical literature has studied biologically distinct products. Muscle-derived myogenic preparations differ fundamentally from multipotent MSC populations derived from bone marrow or adipose tissue, and these differences matter for mechanism, potency, and expected clinical trajectory [8]. Pooling such heterogeneous approaches under a single narrative can inflate expectations while obscuring the reasons why individual strategies underperform.

## Why Translation Stalls: Heterogeneity of Product and Process

A major reason why clinical translation has remained elusive is that the field has not been studying a single, standardized therapy. Product heterogeneity exists at multiple levels. First, the cell source differs (skeletal muscle, bone marrow, adipose tissue), and each source yields cells with distinct differentiation potential, proliferative kinetics, senescence behavior, and secretory profiles [12, 15]. Second, manufacturing and handling vary across studies: tissue processing, culture media, passage number, cryopreservation, thawing protocols, viability thresholds, and phenotyping criteria can all alter product composition and potency [13, 18].

In pharmacology, therapeutic identity is molecularly defined; in cell therapy, “the product” is a complex living population and can drift with process choices. For this reason, modern translational frameworks emphasize standardized characterization, quality control, and validated release criteria. Yet for MSC products in particular, a universally accepted potency assay that predicts clinical efficacy remains lacking. Candidate assays include immunosuppression readouts, cytokine secretion profiling, gene expression signatures, angiogenesis assays, and extracellular vesicle characterization, but these are rarely validated against patient-centered endpoints in SUI [12, 14, 18]. Without potency-linked release criteria, negative or modest results are difficult to interpret: is the problem the biology, the manufacturing, or patient- and tissue-level barriers?

Autologous approaches amplify variability because the donor is the patient. Comorbidities common in SUI populations—obesity, diabetes, metabolic syndrome, chronic inflammation—can alter MSC yield and function and may reduce proliferative and paracrine potency [12, 15]. Even with identical manufacturing protocols, products derived from different patients may differ substantially. This variability complicates trial reproducibility and scale-up and may contribute to heterogeneous clinical response patterns.

Taken together, the heterogeneity of both product and process undermines the comparability of trials, limits the credibility of pooled interpretations, and slows mechanistic optimization.

## Regulatory and Translational Framework: The Advanced Therapy Medicinal Products Challenge

Stem cell–based therapies are regulated as advanced therapy medicinal products in the European Medicines Agency regulatory pathway and as cellular therapy products under the United States Food and Drug Administration biologics and human cells, tissues, and cellular and tissue-based products regulations [20, 21]. These regulatory structures impose essential safety requirements, including donor eligibility and traceability, Good Manufacturing Practice (GMP)–compliant production, sterility and potency testing, viability thresholds, genetic stability assessments, and safeguards against tumorigenicity. Although these standards are necessary to ensure patient safety and product quality, they also introduce substantial complexity, cost, and logistical challenges that may limit trial scalability and slow iterative optimization of therapeutic protocols.

A central regulatory-scientific obstacle is potency. For small molecules, pharmacokinetics and pharmacodynamics provide relatively direct links between product characteristics and clinical effect. For MSC products, potency is multidimensional and context dependent. Regulatory guidance emphasizes the need for potency assays, but there is no consensus on which assays best predict in vivo therapeutic performance in a given indication. For SUI, candidate potency domains include: immunomodulatory capacity (e.g., suppression of activated immune cells), pro-angiogenic signaling, anti-apoptotic effects, and matrix remodeling potential [18, 20]. However, none is routinely validated against continence outcomes, and therefore “release” criteria may not meaningfully stratify products by expected clinical activity.

Regulation also interacts with the choice of autologous versus allogeneic strategies. Autologous approaches may reduce some immunological concerns but amplify variability and manufacturing burden (each patient is a unique batch), whereas allogeneic approaches could improve standardization but raise additional immunological and regulatory questions. Either way, stringent process control is required. GMP production principles for MSCs are well described, yet their implementation varies across centers and trials [13, 18].

Trial methodology is another translational gap. Many SUI cell therapy studies are small early-phase trials designed primarily for feasibility and safety. Regulatory guidance for early-phase cellular therapies underscores careful endpoint selection, dose exploration, and long-term safety surveillance [20]. In SUI, heterogeneity of inclusion criteria (e.g., varying proportions of ISD versus hypermobility), inconsistent endpoints, limited follow-up, and the absence of robust comparator arms have collectively constrained interpretability [8]. For a therapy category aimed at competing with established surgery, the methodological standards of evaluation must be commensurate.

An additional, often overlooked consideration is product comparability across batches and time. “Batch-to-batch consistency” is challenging for living products, and drift in cell phenotype can occur with culture expansion and passage. This has implications for multicenter trials, where harmonization of manufacturing and release testing is essential to avoid center effects being misinterpreted as biological efficacy or failure. Regulatory-grade documentation helps, but it does not substitute for potency assays that are truly mechanistically anchored.

Finally, implementation and economics cannot be ignored. Midurethral slings are relatively low-cost and widely scalable within contemporary surgical practice [3, 19]. Cell therapy manufacturing, storage, and delivery infrastructure is far more resource intensive. Even if modest efficacy were improved, cost-effectiveness would remain a barrier unless meaningful and durable clinical benefit can be demonstrated.

## Target Tissue Biology: A Chronically Remodeled and Potentially Unresponsive Niche

A further explanation for limited clinical impact is that the urethral and periurethral tissues targeted in chronic SUI may not be biologically permissive to robust regenerative remodeling. MSCs are most active when injury-associated chemotactic signals and inflammatory cytokines are present, enabling activation of immunomodulatory and trophic programs [14, 15]. Chronic SUI tissue, in contrast, may exhibit fibrosis, altered ECM architecture, reduced vascularity, and neuromuscular degeneration—features that can blunt regenerative responsiveness. In a quiescent niche, injected cells may fail to receive sufficient activating cues to generate sustained downstream effects [8].

This is not merely a theoretical concern. If the host microenvironment is a dominant determinant of MSC function, then “priming” strategies may be required. Conceptually, priming could involve mechanical or biochemical preconditioning of the periurethral niche, adjunctive growth factors, scaffold-based delivery that enhances retention and local signaling, or repeated dosing paradigms that maintain trophic effects over time. These approaches remain largely investigational in SUI but represent a plausible path toward mechanism-aligned optimization.

Importantly, tissue biology also interacts with patient characteristics. Metabolic disease and systemic inflammation may further limit local repair responses and impair neovascularization. In such contexts, autologous cells may be less potent and the host environment less responsive—compounding constraints at both product and target levels.

## The Biomechanical Imperative: Why Cells Alone May Be Insufficient

Even if cell-based therapies achieve measurable biological effects, continence is fundamentally a biomechanical phenomenon. Stress events impose rapid increases in intra-abdominal pressure, requiring effective pressure transmission and load-bearing suburethral support to prevent leakage. Classical frameworks emphasize the importance of structural support, urethral closure pressure, and the mechanical integrity of pelvic floor and periurethral connective tissues [4, 22, 23].

Midurethral slings succeed largely because they provide immediate biomechanical reinforcement. By restoring suburethral support and optimizing force transmission vectors during stress, slings generate durable clinical benefit [3, 19]. In this context, periurethral injection of cells—without a concomitant load-bearing construct—may be unable to replicate the mechanical effect of a sling, even if biological remodeling occurs. This conceptual mismatch likely explains why clinical improvements are incremental rather than transformative: biological augmentation can modestly improve tissue quality, but it cannot substitute for structural support.

A central implication is that the future of regenerative therapy in SUI may not be as a direct alternative to sling surgery, but as an adjunct or hybrid strategy. Biologically enhanced mechanical support—such as scaffold-based constructs, cell-seeded matrices, or “bioactive” slings—could, in principle, combine immediate mechanical reinforcement with longer-term biologic remodeling. Such an integrated approach aligns mechanism with pathophysiology and provides a clearer pathway to achieving both magnitude and durability of effect.

## Outcome Definitions, Durability, and What Trials Must Do Differently

Outcome heterogeneity remains a practical obstacle. Across trials, “success” has been defined variably: a negative cough stress test, absolute pad weight thresholds, relative improvement on pad tests, symptom score improvements, or composite endpoints [8]. Some definitions prioritize complete dryness; others accept partial improvement. Without harmonized outcomes that are clinically interpretable and patient centered, comparisons across studies are unreliable and perceived efficacy may be inflated by permissive endpoints.

For IUJ readership, the key point is that regenerative therapies must be evaluated with methodological rigor comparable with surgery. This includes standardized definitions aligned with international terminology [1], adequate follow-up to assess durability, prespecified minimal clinically important differences, and transparent reporting of missing data and adherence. Given that cell-based effects may diminish over time if retention is limited, long-term follow-up (≥ 24 months) is essential to establish whether benefit is durable, transient, or dependent on repeated dosing paradigms.

Patient selection is also likely critical. Trials that lump together heterogeneous SUI mechanisms may obscure subtype-specific signals. In particular, ISD-dominant SUI may represent a different biological target than hypermobility-dominant SUI. Mechanism-driven stratification, informed by clinical and urodynamic features, could improve signal detection and guide personalized therapeutic approaches.

## Future Directions: Integration, Priming, and Next-Generation Products

If stem cell therapy is to move beyond feasibility, future development should pivot toward mechanism-driven optimization rather than continued heterogeneity-driven experimentation. Several priorities stand out.

First, product definition and transparency must improve. Standardized characterization (cell identity, purity, viability), process reporting, and potency assays linked to functional biology are essential for reproducibility [13, 18]. Second, delivery strategies must evolve. Approaches that enhance retention (e.g., hydrogels or scaffolds), sustain local signaling (e.g., controlled-release systems), or leverage extracellular vesicles as more stable biologic mediators may be advantageous [9, 17]. Third, priming strategies should be explored to address the biologically quiescent periurethral niche—recognizing that microenvironmental responsiveness is a major determinant of MSC function.

Fourth, integration with biomechanics is likely required. Hybrid strategies—biologically augmented slings, cell-seeded scaffolds, or bioactive support constructs—offer a rational pathway to combining immediate mechanical reinforcement with longer-term biologic remodeling. This may be the conceptual bridge needed to close the gap between biological plausibility and durable clinical efficacy.

Finally, future trials should be designed to answer “why” as well as “whether.” Embedding mechanistic substudies (e.g., imaging of periurethral vascularity, biomarkers of matrix turnover, or standardized measures of urethral function) may help to identify responders, guide dosing strategies, and validate potency assays against clinically meaningful endpoints. Without such mechanistic anchoring, the field risks repeating feasibility studies that confirm safety but do not advance toward durable efficacy.

In parallel, the field should explicitly define the clinical positioning of regenerative interventions. A realistic early target may be symptom reduction in carefully selected patients who decline surgery, or biologic augmentation to reduce recurrence risk after conventional procedures. Clarity on the intended clinical role can sharpen endpoint selection, comparator choice, and sample size assumptions, accelerating progress toward decisions that matter in real practice.

## Clinical Implications

For practicing urogynecologists, the current evidence supports a clear clinical message: stem cell therapy for SUI should be considered investigational. Safety signals in early trials are reassuring [5–7], but efficacy is modest and durability uncertain [8, 18]. Patients should be counseled that established surgical options—particularly midurethral slings—have stronger evidence and more predictable long-term outcomes [3, 4]. Participation in controlled clinical trials is appropriate, but routine clinical adoption outside research settings is premature.

## Conclusion

Stem cell therapy for SUI remains scientifically compelling but clinically incomplete. The persistent gap between biological rationale and clinical performance reflects the heterogeneity of products and manufacturing, the lack of validated potency assays, the regulatory and logistical constraints, a chronically remodeled target tissue that may be biologically quiescent, and—most fundamentally—the biomechanical nature of continence that cell injections alone cannot replicate. Future progress will require integration of regenerative biology with biomechanics, mechanism-driven trial design, and rigorous long-term evaluation. Until then, stem cell therapy should be regarded as a promising hypothesis rather than a definitive solution.
